# Protocol for a scoping review of implementation strategies for integrating evidence-based cardiometabolic disease care for adults into siloed health services in low- and middle-income countries

**DOI:** 10.3389/frhs.2026.1961796

**Published:** 2026-10-16

**Authors:** Amanda Brumwell, Rupal Shah-Rohlfs, Margaret Nickel, Erica Kocher, Mohammed Ali, Ana Olga Mocumbi, Marian Neuhouser, Stephanie Page, Adrienne Shapiro, Kenneth Sherr, Sarah Gimbel

**Affiliations:** 1 Department of Global Health, School of Public Health, University of Washington, Seattle, WA, United States; 2 Heidelberg Institute of Global Health, Medical Faculty and University Hospital, Heidelberg University, Heidelberg, Germany; 3 Emory Global Diabetes Research Center, Woodruff Health Sciences Center, Emory University, Atlanta, GA, United States; 4 Hubert Department of Global Health, Emory University Rollins School of Public Health, Atlanta, GA, United States; 5 Department of Family and Preventive Medicine, Emory University School of Medicine, Atlanta, GA, United States; 6 Faculty of Medicine, Universidade Eduardo Mondlane, Maputo, Mozambique; 7 Cancer Prevention Program, Fred Hutchinson Cancer Center, Cancer Prevention Program, Seattle, WA, United States; 8 Department of Epidemiology, School of Public Health, University of Washington, Seattle, WA, United States; 9 Diabetes Institute, School of Medicine, University of Washington, Seattle, WA, United States; 10 Department of Metabolism, Endocrinology, and Nutrition, School of Medicine, University of Washington, Seattle, WA, United States; 11 Division of Allergy and Infectious Diseases, School of Medicine, University of Washington, Seattle, WA, United States; 12 Department of Child, Family, and Population Health Nursing, School of Nursing, University of Washington, Seattle, WA, United States

**Keywords:** cardiometabolic disease, implementation outcomes, implementation science, implementation strategies, integrated care, low- and middle-income countries, noncommunicable diseases, scoping review

## Abstract

**Background:**

Cardiometabolic diseases, including type 2 diabetes, hypertension, obesity, and dyslipidemia, represent a growing burden in low- and middle-income countries (LMICs), where health systems have historically prioritized vertically organized infectious disease programs. While integrated care models co-locating cardiometabolic and infectious disease services have been increasingly documented, the implementation strategies used to design, deploy, and sustain these integrations have not been systematically characterized.

**Objective:**

This scoping review aims to identify, characterize, and map the implementation strategies used to integrate evidence-based cardiometabolic disease care into established HIV, tuberculosis, and primary care platforms in LMIC settings.

**Methods:**

We will conduct a structured search across five databases (PubMed, Embase, Cochrane Library, Web of Science, and CINAHL) following the methodological frameworks of Arksey and O'Malley and the Joanna Briggs Institute, and reporting in accordance with PRISMA-ScR guidelines. Eligible studies will undergo staged screening and data extraction by a team of trained reviewers, and the methodological quality of included studies will be appraised using the Mixed Methods Appraisal Tool (MMAT). Implementation strategies will be classified using the Expert Recommendations for Implementing Change (ERIC) framework; implementation context will be characterized to assess the setting, population, and circumstances of implementation; and implementation outcomes will be systematically assessed and characterized using the Proctor et al. taxonomy.

**Expected outputs:**

Findings will be synthesized as a narrative and tabular evidence map characterizing the implementation strategies, implementation outcomes, evidence-based interventions, and contextual factors associated with cardiometabolic care integration in LMICs, with the aim of informing future implementation research, programmatic implementation, health systems reorganization, and policy development.

**Clinical Trial Registration:**

This protocol was registered prospectively on the Open Science Framework (https://doi.org/10.17605/OSF.IO/2E89M).

## 1 Introduction

Chronic, non-communicable diseases are increasing globally, contributing to substantial morbidity and mortality (1, 2). Chief among these conditions are cardiometabolic diseases (CMD), a category of closely-related and frequently co-occurring conditions affecting the cardiovascular, endocrine, and renal systems, among others (3, 4). CMDs, including cardiovascular disease and type II diabetes (T2D), are leading causes of death and morbidity worldwide. The burden is predicted to grow in the coming decades, particularly in low- and middle-income countries (LMICs) (2, 5–8). These conditions require lifelong continuity of care to support disease management and mitigate risk of severe complications. Effective management of blood pressure, blood glucose, obesity, and dyslipidemia, which are among the most prevalent manifestations of CMDs, can prevent adverse outcomes, including myocardial infarctions, strokes, and chronic kidney disease (9–16). Globally, an estimated 1.28 billion adults live with hypertension, 589 million adults live with diabetes, and more than one billion adults live with obesity; low- and middle-income countries are home to the greatest proportion of these and the greatest increases in the occurrence of these conditions (7, 8, 17). Dyslipidemia is a leading risk factor for cardiovascular mortality, and it includes hypertriglyceridemia and hypercholesterolemia, of which the global prevalence is estimated to be 24.1% and 28.8% respectively, although dyslipidemia burden is unevenly quantified in low and middle-income settings (18). Together, T2D, hypertension, obesity, and dyslipidemia account for a large and growing share of the disability-adjusted life years attributable to noncommunicable disease globally, but particularly in in low- and middle-income countries (1). Effective treatments for each of these four conditions are well established, yet there remains a substantial gap in the delivery of these treatments (19, 20). Closing this gap in low- and middle-income settings requires innovation in the ways in which treatment is delivered, adapted, scaled, and sustained in health systems that were organized around other priorities, such as infectious diseases. Effective and efficient implementation strategies are urgently needed to address the increasing burden of these four conditions at the population-level.

In many LMIC settings, health systems lack the capacity to prevent, detect, and treat CMD, despite gains in health system capacity in recent years. This is because health system improvements have largely been driven by policies and funding that prioritized individual diseases and conditions, most notably infectious diseases like HIV and tuberculosis (TB), as well as maternal and child health (21, 22). However, these services tend to be vertically aligned, wherein services delivered across the spectrum from the community level to referral hospitals are primed to deliver care for a specific disease or condition, and major health system components (such as but not limited to consumables, human resources, laboratory and diagnostic systems, information systems, and funding) are oriented to the goal of eliminating a particular disease (23, 24). As a result of these vertical systems and the major funding and governance powers that have precipitated these systems’ priorities, other diseases and their prevention have fallen by the wayside. This is particularly salient in the case of T2D and other CMD, whose onsets are slow, with etiology driven by overlapping socioeconomic and behavioral drivers, and with cross-cutting harms to the human body that are not easily addressed within a vertically-oriented health system.

Recently, numerous efforts have been made to leverage health systems’ established services for HIV and TB to address cardiometabolic conditions, including T2D and HTN (25–28). Much of the published evidence on integrated care originates from sub-Saharan Africa, which weights the evidence base towards this region, yet similar approaches have been documented across diverse LMIC contexts, including in South and Southeast Asia and Latin America, underscoring the broader global relevance of this health system strategy (29–32). It is therefore important to characterize evidence of integration across different regions. These models of integrated care may be advantageous due to the epidemiologic overlap between HIV and HTN (32–34, 70) and between TB and T2D (35, 36). Moreover, obesity and dyslipidemia are additional cardiometabolic conditions that frequently co-occur with T2D and HTN and are themselves established risk factors for cardiovascular disease, but these have received comparatively less attention among programs integrating cardiometabolic and infectious disease care (37, 38). Given that HTN and T2D are co-occurring, that evidence is emerging indicating the acceptability and feasibility of integrating care (27, 39–42), and that recent changes in international aid and biomedical research policies have halted infectious disease programs that relied on external support (43–45), we may be at a critical juncture to rethink the organization of health services in ways that may improve the quality, efficiency, and resilience of care.

Despite growing interest in models for integrated cardiometabolic and infectious disease care, evidence-based interventions (EBI) and implementation strategies for doing so are not well defined. A recent scoping review of strategies used to integrate noncommunicable disease services into HIV and tuberculosis platforms in low- and middle-income countries found that fewer than a third of included studies described a fully integrated service delivery model, and that the strategies reported clustered narrowly around training, evaluation, and provider assistance, which suggests that strategies remain thinly specified even where integration itself has been documented (46). Our review extends that work by adding obesity and dyslipidemia, by treating primary care as a service platform of equal interest alongside HIV and tuberculosis, and by specifying each strategy at the level recommended by Proctor and colleagues rather than counting strategy categories (47). Established infectious disease platforms are a logical foundation for this integration because they already provide the longitudinal, chronic-care infrastructure that the ongoing management of cardiometabolic conditions also demands, including routine follow-up, patient retention and tracing systems, decentralized medication distribution, and recurring clinical contact. Prior systematic reviews of HIV- and tuberculosis-noncommunicable disease integration have documented the feasibility and acceptability of leveraging these systems for cardiometabolic care (26, 48, 49). To enable a thoughtful expansion of integrated services that are well-suited to meet communities’ dual burdens of chronic and infectious diseases, it is important to understand the best practices for such integration. In addition, it is necessary to identify the distinctive barriers and facilitators for integrating care for T2D, HTN, obesity, and dyslipidemia, which may differ from those occurring in existing siloed programs like those for infectious diseases.

The objective of this scoping review is to identify and describe implementation strategies used to integrate evidence-based practices for T2D, HTN, obesity, and dyslipidemia care for adults into discrete health services in LMIC settings. We concentrate on these four conditions, rather than on cardiometabolic disease more broadly, because services for these conditions can be reasonably delivered at the primary healthcare level. Severe conditions related to CMD (e.g., stroke, metabolic dysfunction-associated steatotic liver disease, and myocardial infarction) typically require specialized or emergency care, which meaningfully differ from the established service platforms into which routine care for these conditions might be feasibly integrated. We will conduct a scoping review of published literature to identify the actions taken by policymakers, health systems, implementers, and health care providers in LMICs to deliver care for T2D, HTN, obesity, and dyslipidemia within primary healthcare services and infectious diseases programs.

We have selected HIV, TB, and primary care services as the service platforms of interest due to the proportion of international and domestic funding devoted to these systems and due to their primarily adult patient population. Notably, we consider community-based initiatives to fall within the scope of primary care services, given the proximity of primary care to the communities it serves, the established roles that community health workers provide in bridging communities to low-level health services, and the subsequent difficulty in differentiating between facility- and community-based care. Whereas TB and HIV systems have received attention as potential integrative platforms for CMD services (49–52), primary care is the only one of the three platforms whose core mandate already includes chronic disease care, and one that reaches adults who are not enrolled in any disease-specific program. Therefore, we might expect that integration efforts may deploy different strategies compared to those used in infectious diseases platforms. Although these platforms differ in the typical duration and intensity of care they provide, ranging from time-limited tuberculosis treatment, to lifelong HIV care, to the broad and longitudinal scope of primary and community-level, each is an established, adult-facing service platform offering the workforce, retention systems, and routine patient contact that the delivery of chronic cardiometabolic care requires. We therefore treat these structural differences across platforms as strata for analysis rather than assuming the platforms are equivalent.

We will extract information regarding EBIs, implementation strategies and implementation outcomes including effectiveness, and we will synthesize this information to identify research gaps and priorities for future policies and practice. By the end of this review, we will have identified programmatic components that have been deployed to extend, improve, or make more efficient the delivery of care for conditions related to CMD within vertical health services.

## 2 Methods and analysis

We will conduct a scoping review due to the exploratory nature of our investigation. As suggested by Arksey and O’Malley, a scoping review is suited to mapping the size and scope of available evidence and research rigorously and transparently (53). Our review will leverage the steps recommended by the Joanna Briggs Institute and by Arksey and O’Malley, which include the following components: 1) identifying the research question, 2) identifying relevant studies, 3) selecting eligible studies, 4) charting the data, and 5) summarizing results (53–55). We will leverage the PRISMA-ScR checklist to ensure that our methods align with standard reporting practices (Supplementary Material) (56).

### 2.1 Research question

Our primary research question is “What are the strategies used to integrate type II diabetes, hypertension, obesity, and dyslipidemia care into established care delivery platforms for non-pregnant adults in low-/middle-income settings?” We have identified the following sub-questions to further guide our search:

1) What core programmatic activities were undertaken, and by whom, to integrate care for cardiometabolic conditions (T2D, HTN, obesity, and dyslipidemia) among adults with established care platforms including those for HIV, TB, and primary care?
2) What were the conditions (i.e., the setting, population, and circumstances) where these activities were and were not effective? (57)
3) What implementation strategies and adaptations were used to improve the delivery of core integrative activities?

### 2.2 Identifying relevant studies

We will employ a two-step search strategy, prioritizing peer-reviewed published literature, to identify articles for inclusion, and we will search in the following databases: PubMed, Embase, Cochrane Library, Web of Science, and CINAHL. First, we will search each of the databases for articles pertaining to the study questions, performing a review of article titles, text, and Medical Subject Headings (MeSH) or keywords that are inclusive of the following terms: “hypertension” or “type II diabetes” or “obesity” or “dyslipidemia”, “integration” or “integrated care”, and “HIV” or “tuberculosis” or “TB” or “primary care”. Second, we will refine the search string based on the key terms identified in the preliminary search, and this search string will be tailored for each of these databases. These conceptual term groups are illustrative rather than the executable query; the final search string for each database will be developed in collaboration with a health sciences librarian, using database-specific controlled vocabulary (e.g., MeSH and Emtree) and explicit Boolean structure with appropriate nesting and parentheses to combine the cardiometabolic conditions of interest (including obesity and dyslipidemia), the integration concepts, and the care platforms. The strategy will include a fourth group of search terms for setting, leveraging a search filter for low- and middle-income countries using the World Bank country name terms and regional terms, so that LMIC settings are identified through the search itself rather than only at screening. This list will be generated using the World Bank's classification for countries’ income status. The string for each database will be validated against the benchmark set described below before the search is finalized.

We will employ a search validation procedure prior to finalizing our search string. A benchmark set of known, relevant articles will be identified through preliminary scoping of the literature by the first author and reviewed by the investigative team for confirmation that each article clearly meets our inclusion criteria. This benchmark set will aim to include articles that vary in their geographic setting, care platform (HIV, TB, or primary and community-level care), and cardiometabolic condition of focus, in order to test whether the search string performs adequately across the conceptual breadth of the review. We anticipate identifying approximately five to ten anchor articles for this purpose.

The final search string will be tested against this benchmark set across each database independently, as controlled vocabulary and indexing conventions differ across platforms. If a benchmark article is not retrieved by a given database, we will examine whether this reflects a limitation of that database's indexing rather than a deficiency in the search string before making adjustments. The string will be refined iteratively until all benchmark articles are successfully retrieved, or until any gaps are attributable to database-level indexing limitations rather than search string deficiencies. The final validated search string, along with the number of results returned per database and the date of the search, will be documented in Table 1 of the protocol. If the validated search yields fewer than 20 eligible articles at full text, we will implement a pre-specified expansion in the following order: we will lower the integration threshold to include linkage, and we will manually search the references of included articles and of relevant review articles. Any such expansion will be documented and reported as a deviation from the registered protocol.

**Table 1:** Search strategy log.

No.	Database	Search string used	Number of citations returned	Date
1	*Example:* PubMed	*Example:* (“diabetes” OR “type II diabetes” OR “T2D” OR “DM2”) OR (“hypertension” OR “HTN”) AND (“integrat*”) AND ((“primary care”) OR (“HIV” or “human immunodeficiency virus”) OR (“tuberculosis” OR “TB”)	*Example: 340*	*Example: June 20, 2025*
2	*Example:* Embase			
3	*Example:* Cochrane			
4	*Example:* CINAHL			

We will maintain a log of the searches employed using the template provided in Table 1. Identified articles will be managed in Covidence, and duplicates will be removed prior to article review.

For the purposes of this aim, and leaning on well-established criteria from the literature, EBIs will be defined as the programs, practices, processes, policies, and guidelines with a demonstrated level of effectiveness to improve health outcomes, services, or systems (57, 58). In practice, an intervention will be considered evidence-based if it is recommended in a World Health Organization or national clinical guideline, if the article cites evidence of its effectiveness, or if it appears in established guidelines for evidence-based interventions. We expect most included articles to satisfy this criterion, because the clinical content of cardiometabolic care that can be delivered at the primary healthcare level is largely guideline-based. Strategies will be defined as the “methods or techniques used to enhance the adoption, implementation, and sustainability” of the EBIs identified (59, 60). Adaptations will be defined as the deviations and changes made to the EBIs or to the strategies selected for EBI implementation with the intention to increase their suitability to the context in which they are being delivered. We will follow the PRISMA guidelines for reporting scoping reviews to select, review, and synthesize articles in this aim, and we will use Covidence to manage the articles and resources identified in this search (61, 62).

### 2.3 Selection of eligible studies

#### 2.3.1 Title and abstract screening

We will screen each of the identified articles’ titles and abstract for relevance. A team of four reviewers will conduct the article title and abstract screening, applying inclusion and exclusion criteria described below. Each article will be screened by two reviewers. The reviewers will first screen a random selection of 10% of the articles, aiming for ≥85% agreement. Inter-rater reliability for this calibration sample will be quantified using Cohen's kappa and reported alongside the percent agreement. They will reach consensus on any discordantly screened articles with the assistance of a third reviewer from the screening team. If they achieve ≥85% agreement, they will proceed with double screening the remaining articles. If there is <85% agreement in the initial calibration, the reviewers will review the screening criteria, make necessary calibrations to improve clarity, and dually screen another random 10% of the identified articles, aiming for ≥85% agreement. After ≥85% agreement is achieved, the reviewers will dually screen the remaining articles and again aim for ≥85% consensus. After this calibration period, discordantly screened articles will be reviewed for inclusion by a third screener; the review team will discuss and reach consensus on discordant articles as needed.

#### 2.3.2 Inclusion criteria

This review will apply the Population–Concept–Context (PCC) framework outlined by the Joanna Briggs Institute (JBI) to guide study selection in a systematic and transparent manner. The inclusion criteria have been developed to align with the overall objective of the review, which is to identify and map initiatives that aim to implement or improve the quality of health services addressing type II diabetes, hypertension, obesity, or dyslipidemia in low- and middle-income countries (LMICs).

##### 2.3.2.1 Population

The review will include studies involving adults aged 18 years or older who are non-pregnant and receiving care for one or more of the following chronic conditions: type II diabetes mellitus, hypertension, obesity, or dyslipidemia. Studies that examine health care providers, caregivers, or community health workers will also be included when their roles are directly related to the delivery, implementation, or improvement of services for these conditions.

##### 2.3.2.2 Concept

The central concept of the review is the implementation or enhancement of health service delivery for the specified chronic conditions. Eligible sources must describe a programmatic or research initiative that seeks to implement, evaluate, or improve the quality of health services for type II diabetes, hypertension, obesity, or dyslipidemia. Included studies may report on completed interventions, ongoing programs, or protocols for planned initiatives. Articles presenting qualitative, quantitative, mixed-methods, or economic analyses findings will be eligible when they contribute to understanding the processes, outcomes, or contextual factors associated with implementation or service improvement. Articles focusing solely on literature reviews, commentaries, or opinion pieces will be excluded.

We define implementation as one or more deliberate actions taken to support the adoption, delivery, adaptation, or sustainment of care for hypertension, T2D, obesity, or dyslipidemia. An article is eligible for inclusion at this stage if it describes such an action, which may include, but is not limited to, provider training, task shifting, health information system modifications, redesign of clinical workflows, decentralization of care, and program scale-up. We will not require that an article explicitly identifies itself as an implementation study, use implementation science terminology, or report implementation outcomes, as we expect that integration efforts in these settings may be reported as programs, pilot projects, quality improvement activities, or ministry-led service initiatives, all of which may lack implementation science-focused terminology or framing. Restricting our search to articles explicitly using this language may inadvertently exclude meaningful evidence. Articles for which it is not possible to determine from the title and abstract whether such an implementation action is described will be carried forward to full text review.

##### 2.3.2.3 Context

The review will consider studies conducted in countries classified as low- or middle-income according to the World Bank's income classification at the time of publication. Contexts of interest include a range of health system levels and settings, such as community-based, primary care, and hospital environments. Information on geographic location, health system characteristics, and sociocultural factors will be extracted to support interpretation and mapping of the evidence.

#### 2.3.3 Exclusion criteria

Studies will be excluded if they do not focus on type II diabetes, hypertension, obesity, dyslipidemia, or other chronic cardiometabolic conditions as the primary health condition targeted for service improvement. Articles will also be excluded if they describe programs or research conducted outside of LMIC settings, report only epidemiological characteristics or etiological relationships without reference to care delivery, or focus exclusively on children, adolescents, or pregnant and perinatal populations (including gestational diabetes, pre-eclampsia, or eclampsia). Studies that are not published in English, lack an available abstract or full-text report, or do not present original or programmatic research (e.g., literature reviews, systematic/scoping reviews, or narrative reviews) will also be excluded.

Articles that describe broader cardiometabolic disease services without specifying HTN or T2D care will be included for full-text review. Articles whose relevance is not certain after title and abstract review will be included for full-text review. Due to anticipated ambiguity in articles’ reporting of the implementation context, particularly when cardiometabolic services are integrated into primary health care settings, we will not consider whether services were integrated into TB, HIV, or primary care during the title and abstract screening stage.

#### 2.3.4 Full text review

Articles that meet the screening criteria, or for which eligibility is unclear after title and abstract screening, will undergo full text review by the review team. A random selection of 10% of included articles will be doubly reviewed. The reviewers will aim to achieve ≥90% agreement in their review and will reach consensus on any discordantly judged articles with the assistance of a third reviewer. Agreement for the calibration sample will be reported as both percent agreement and Cohen's kappa. If there is <90% agreement in this initial review, the authors will revise the eligibility criteria as needed, and apply this criteria on another 10% of the articles until ≥90% agreement is reached. The reviewers will then randomly divide the remaining articles, each conducting full text review for the remaining articles such that each article will be reviewed once. As a quality check, a random 10% of singly-reviewed articles will be independently reviewed by a second reviewer; agreement for this sample will also be reported. All discordant decisions (whether from the calibration sample or the quality check sample) will be resolved through discussion, with a third senior reviewer consulted if consensus cannot be reached.

Intentionally taking a broad scope, our eligibility criteria will include: (1) English-language study protocols, peer-reviewed study results, and programmatic and evidentiary reports; (2) articles that include a description of type II diabetes, hypertension, obesity, or dyslipidemia services integrated within HIV, tuberculosis services, or primary care services with the intent to improve cardiometabolic health outcomes; (3) articles that describe, at a minimum, an actor and action undertaken to integrate care per Proctor et al’s strategy specification; (4) articles that include a description of at least one EBI that was delivered as a part of the study or program it reports; and (5) articles that report on efforts undertaken in LMIC settings.

To define and characterize service integration consistently across a heterogeneous evidence base, integration will be defined using two established frameworks, which were identified among Piquer-Martinez et al's scoping review of theories, models and frameworks for health systems integration (63). The first framework by Leutz will be used to determine the depth of integration, wherein each example of integration will be classified as linkage, in which services remain separate and patients are referred between them; coordination, in which services remain structurally separate but share protocols, records, scheduling, or staff time; or full integration, in which a single team delivers both services in one visit and one record (64, 65). Articles eligible for full text extraction will report on implementation at the level of coordination or above, which excludes referral and co-location alone. We will record the assigned level as an extracted variable. To describe the content of integration, we will record which critical health system functions were affected by the integrative initiative; these functions are described by Atun and colleagues and include governance, financing, planning, service delivery, monitoring and evaluation, and demand generation (66).

We will exclude articles during this full text review for which (1) an English-language, full-text article is unavailable, (2) the care delivery platform into which diabetes or hypertension services are integrated is not described, (3) type II diabetes, hypertension, obesity, or dyslipidemia services, service delivery, or outcomes were not the focus of the article, (4) type II diabetes, hypertension, obesity, or dyslipidemia services were not integrated into an established care platform, (5) no EBIs are described, or (6) the article does not report results in LMICs, (7) no deliberate action taken to establish, deliver, adapt, or sustain the integrated service is described, or (8) an action is described but the text does not permit both the actor and the action to be identified.

### 2.4 Charting the data

Data extraction will be conducted by human reviewers in three stages: a form development and piloting stage, a calibration stage, and a final extraction stage. No AI or automated extraction tools will be employed.

#### 2.4.1 Form development and piloting

The data extraction form will be developed by the first author based on the variables of interest and built within Covidence. Prior to full extraction, the form will be piloted by all reviewers on a small sample of five to ten included articles. Reviewers will extract independently and then compare responses to identify ambiguities, missing fields, or inconsistencies in how extraction items are interpreted. The form will be revised iteratively until the team reaches consensus on all field definitions and decision rules. Piloting will be conducted by human reviewers.

#### 2.4.2 Calibration

Following form finalization, reviewers will independently extract a random 10% sample of included articles in duplicate to assess inter-extractor agreement. For structured and categorical extraction fields, agreement will be assessed using percent agreement and, where the number of categories permits, Cohen's kappa. For narrative and qualitative fields, which are not amenable to kappa, agreement will be assessed by comparing extractors’ entries side by side for concordance in meaning and coding against the agreed field definitions, with discrepancies adjudicated through team discussion. Any discordant extractions will be discussed as a group and used to further refine field definitions or add clarifying guidance to the extraction form. Extraction will not proceed to the full sample until the team is satisfied with the level of agreement achieved during calibration.

#### 2.4.3 Full extraction

Remaining articles will be divided among reviewers for single extraction. Single extraction with a duplicated quality-control subset, rather than full double extraction, was chosen to balance reviewer capacity against the risk of extraction error; the preceding calibration step, the standardized extraction form, and the 10% double-extraction check are intended to mitigate this risk. As a quality check, a random 10% of singly-extracted articles will be independently extracted by a second reviewer. Discordant extractions identified through the quality check will be resolved through discussion, with a third reviewer consulted to mediate if consensus cannot be reached. The data to be extracted from each included article will include: study or program characteristics (e.g., location, year, and duration), its purpose (e.g., stated objective, cardiometabolic services that were subject to integration, care platform into which integration occurred), methods (e.g., study or program design, data collection methods, and data analysis, if any), study outcomes (e.g., findings, conclusions, and magnitude of health outcomes), and implementation details (e.g., the EBI; any implementation strategies deployed; adaptations made to the EBI and/or implementation strategies; and implementation conditions that inform the selection of the EBI or implementation strategies or adaptations to either) (Table 2). We anticipate that there will be heterogeneity in the details provided regarding EBIs, implementation strategies, and their adaptations. For this reason, reviewers will code strategies based only on the description within an article, and will not infer an unstated strategy based on the implementation setting or its plausibility. The extraction form will include a field to record a verbatim quotation in support of each coded implementation strategy, as well as a field to record whether the mapping to the ERIC taxonomy was explicit or inferred. We will rely on the definitions of EBIs, implementation strategies, and adaptations that are provided in Section 2.2. We will further characterize integration using Atun et al's framework for health systems integration to identify components of the health system that are affected by integrative initiatives, as described in Section 2.3.4. We will also record whether implementation outcomes are reported, although we will not require that they be explicitly reported or named. These will be identified from studies’ aims, design, and measures, and they will be mapped to Proctor et al's taxonomy of implementation outcomes (67). If multiple articles report on the same study or initiative, data from these articles will be consolidated.

**Table 2:** Key data points for eligible article review.

Variable for collection	Details
Article title	
Publication journal	
Publication year	
Authors	
Name of study/initiative	
Purpose of study	
Year(s) of implementation	
Location (Country, Province/State, City/Town/District)	
Population and sampling method	
Study/program design	
Data Collection methods	
Data analysis	
Study findings/conclusions	
Description of and magnitude of outcome of integration	*As available, the impact of the EBI and implementation strategy on population and/or individual-level health outcomes.*
NCD topic of interest	
Component(s) of NCD control that were integrated: identification of disease, treatment and management of disease, and/or prevention of disease	
Health program into which NCD care was integrated	
Specific EBI(s) implemented	*The programs, practices, processes, policies, and guidelines with a demonstrated level of effectiveness to improve health outcomes, services, or system.*
Integration depth	*Per Leutz, integration will be categorized as coordination or full integration. Articles describing the integration efforts that only reflect linkage, the lowest tier of integration, will be excluded from full text review, and will have this integration depth noted as a reason for exclusion for reporting purposes. (* 64 *)*
Integration components	*Per Atun et al, the components of the health system affected by integration will be documented as governance, financing, planning, service delivery, monitoring and evaluation, and demand generation.* (66)
Implementation strateg(ies)	*Methods or techniques used to enhance the adoption, implementation, and sustainability of the evidence-based interventions identified. Key data will include, as available, a description of the action, actor, action target, temporality, dose, implementation outcome affected, and justification for selected strategy.* (59)
Implementation outcomes and measures	*Implementation outcomes reported for the integration effort, defined following the taxonomy of Proctor and colleagues as the effects of deliberate actions to implement an EBI, distinct from the health service and patient outcomes those actions are intended to improve. Key data will include, as available, the outcome(s) assessed (acceptability, adoption, appropriateness, cost, feasibility, fidelity, penetration, or sustainability), the level at which each was assessed, the measure or indicator used, and the reported result.* (67)
ERIC Strategy Mapped	
Adaptations to EBI or IS	*Modifications made to EBI or implementation strategy, either planned proactively as part of the program or study's intentional design, or made in response to contextual factors, such that the EBI and/or strategy is more appropriately tailored to the implementation context, and reason for this adaptation.*
Implementation conditions	*The setting, population, and circumstances in which the EBI and strategy were implemented and which informs adaptations to the EBI or IS.*

### 2.5 Critical appraisal of included studies

To assess the methodological quality of the included studies and to strengthen interpretation of the evidence, each included study will be critically appraised using the Mixed Methods Appraisal Tool (MMAT, version 2018) (68). The MMAT was selected because our inclusion criteria span qualitative, quantitative (randomized, non-randomized, and descriptive), mixed-methods, and economic study designs, and it provides a single validated instrument for appraising this range of designs against design-specific criteria. After a calibration period on a subset of the included articles, two reviewers will independently appraise each included study, resolving discordant ratings through discussion and, where consensus cannot be reached, through consultation with a third reviewer. Consistent with scoping review methodology and with MMAT guidance, appraisal results will be used to characterize the methodological strengths and limitations of the evidence base and to contextualize the synthesis, rather than as a threshold for excluding studies. The distribution of appraisal ratings will be summarized narratively and in tabular form, including in relation to study design and to the implementation outcomes assessed.

### 2.6 Collating, summarizing, and reporting results

We will extract and summarize information regarding the EBIs utilized, the strategies for implementation, any adaptations made and whether these were proactively designed or reactively deployed, and the context in which the EBIs and their strategies were implemented. This information will be reported as a table in the final manuscript. We will also note the magnitude of effectiveness on health outcomes for each article's EBI and strategy, should that information be available. We will qualitatively assess the conditions of implementation and identify any patterns in contextual conditions and the EBI's effectiveness, or lack thereof, of the intervention. We will describe the strategies used to deliver EBIs in each of the articles, in terms of the actor, action, action target, temporality, dose, implementation outcome affected, and justification for those strategies, as well as adaptations to those strategies and the reasons for those adaptations (59). Finally, we will map these strategies to the strategies defined in the Expert Recommendations for Implementing Change (ERIC) framework (60). For strategies that lack analogues in the ERIC Framework, these and their distinguishing differences from ERIC strategies will be clearly documented and reported as potential innovations for integrating care. Given the anticipated heterogeneity in study designs, settings, and reporting, we will not conduct a meta-analysis; instead, we will manage this heterogeneity by charting included study against common frameworks (the PCC elements, the ERIC strategy taxonomy, and Proctor and colleagues’ implementation outcomes) and by organizing the narrative synthesis around care platform, cardiometabolic condition, and strategy type, so that diverse studies can be compared along consistent dimensions. We will also report the geographic distribution of included studies, the distribution of integration depth, the proportion of ERIC mappings that were inferred rather than explicit, and the number of articles excluded at full text and the rationale for their exclusion.

This protocol has been registered on the Open Science Framework (OSF) prior to data collection [Registration DOI 10.17605/OSF.IO/2E89M] (69).

## 3 Discussion

The identification of implementation strategies used to deliver EBIs will fill a clear gap in the knowledge base, moving the needle on research that has predominantly focused and reported on the effectiveness of disease care integration rather than the design of programs and strategies to better deliver evidence-based care (50). This gap limits the ability of policymakers and program designers to translate existing evidence into actionable, context-appropriate guidance for health systems reorganization. By clearly synthesizing EBIs, strategies, and the contexts in which those strategies did or did not affect improvements in chronic disease outcomes, we will generate evidence to inform future efforts that seek to design and scale better chronic disease control initiatives.

We anticipate some limitations in this study. First, because implementation science and the discourse around integrated care are both relatively recent, we cannot anticipate the size of the eligible evidence base, and we may find either that few articles meet our criteria or that eligible articles describe their implementation strategies in limited detail. Our eligibility criteria were intentionally defined broadly to mitigate the first scenario, and we will consider expanding them if we identify too few eligible articles. For the second scenario, we will report the completeness with which implementation strategies are described as a finding of the review rather than treat it as a deficiency of our search. Second, in our effort to define a realistic and informative research question, we are limiting our scope to four cardiometabolic conditions (T2D, HTN, obesity, and dyslipidemia), rather than the broader categories of cardiovascular or chronic disease. As a result, this scoping review may not identify EBIs or implementation strategies that have been leveraged for other types of chronic disease (e.g., mental health conditions) that are unrelated to cardiometabolic disease but are nonetheless informative for improving cardiometabolic disease care. Third, maternal, child, and perinatal health services also receive a substantial amount of funding in LMICs compared to funding for TB, HIV, and primary care settings. Although screening and care for cardiometabolic conditions among pregnant women including but not limited to gestational hypertension, preeclampsia, and gestational diabetes tend to be a core component of perinatal health services, we expect integration strategies for cardiometabolic services to be different, with limited transferability to other health platforms. Therefore, we recommend that a separate review be conducted to better understand EBIs and implementation strategies for these perinatal conditions. Fourth, by restricting eligibility to studies published in English, this review may be subject to language bias and may underrepresent integration efforts reported in other languages; we accept this constraint for feasibility but acknowledge that it may limit the comprehensiveness of the evidence map. Finally, although scoping reviews do not typically include a formal quality appraisal, we will appraise the methodological quality of included studies using the Mixed Methods Appraisal Tool to strengthen interpretation of the evidence. Because this appraisal is intended to characterize rather than to exclude studies, some heterogeneity in study rigor will remain, and the resulting evidence map should be interpreted as a synthesis of which implementation strategies have been used and in what contexts rather than as a graded assessment of intervention effectiveness.

## 4 Ethics and dissemination

This review uses previously published literature and does not involve human participants, their identifiable data, or animals, and institutional ethics approval and informed consent were therefore not required. The protocol was registered prospectively on the Open Science Framework (https://doi.org/10.17605/OSF.IO/2E89M) before data collection, and any deviations from the registered protocol will be documented and reported in the resulting manuscript.

We will disseminate the findings of this review through publication in a peer-reviewed journal and through presentation at implementation science and global health scientific meetings. The completed charting outputs, including the full evidence table, will be made available as Supplementary Material alongside the resulting article. We will also share a summary of the evidence map with ministry of health and implementing partner audiences in the settings in which our team works, so that the findings can inform decisions about integrating cardiometabolic care into established service platforms.
